# Dissociated Neurons and Glial Cells Derived from Rat Inferior Colliculi after Digestion with Papain

**DOI:** 10.1371/journal.pone.0080490

**Published:** 2013-12-12

**Authors:** Odett Kaiser, Pooyan Aliuos, Kirsten Wissel, Thomas Lenarz, Darja Werner, Günter Reuter, Andrej Kral, Athanasia Warnecke

**Affiliations:** Department of Otolaryngology, Hannover Medical School, Hannover, Germany; Glasgow University, United Kingdom

## Abstract

The formation of gliosis around implant electrodes for deep brain stimulation impairs electrode–tissue interaction. Unspecific growth of glial tissue around the electrodes can be hindered by altering physicochemical material properties. However, in vitro screening of neural tissue–material interaction requires an adequate cell culture system. No adequate model for cells dissociated from the inferior colliculus (IC) has been described and was thus the aim of this study. Therefore, IC were isolated from neonatal rats (P3**_**5) and a dissociated cell culture was established. In screening experiments using four dissociation methods (Neural Tissue Dissociation Kit [NTDK] T, NTDK P; NTDK PN, and a validated protocol for the dissociation of spiral ganglion neurons [SGN]), the optimal media, and seeding densities were identified. Thereafter, a dissociation protocol containing only the proteolytic enzymes of interest (trypsin or papain) was tested. For analysis, cells were fixed and immunolabeled using glial- and neuron-specific antibodies. Adhesion and survival of dissociated neurons and glial cells isolated from the IC were demonstrated in all experimental settings. Hence, preservation of type-specific cytoarchitecture with sufficient neuronal networks only occurred in cultures dissociated with NTDK P, NTDK PN, and fresh prepared papain solution. However, cultures obtained after dissociation with papain, seeded at a density of 2**×**10^4^ cells/well and cultivated with Neuro Medium for 6 days reliably revealed the highest neuronal yield with excellent cytoarchitecture of neurons and glial cells. The herein described dissociated culture can be utilized as in vitro model to screen interactions between cells of the IC and surface modifications of the electrode.

## Introduction

Neurostimulation through implanted electrodes is routinely used to alleviate symptoms of neurological disorders including Parkinson's disease, epilepsy, essential tremor, dystonia, and psychiatric disorders [1], [2]. Within the auditory system, electrical stimulation can be used in order to elicit hearing sensation. The success achieved by the electrical stimulation of the peripheral auditory system via a cochlear implant (CI) [3]–[5] encouraged for the development of strategies for the hearing restoration in patients with retrocochlear damage. Auditory brainstem implants (ABI) and the penetrating auditory brainstem implants (PABI) are used to stimulate the cochlear nucleus (CN) [6], [7], however with limited performance [8]–[12]. The lack of success after treatment of neurofibromatosis type II patients with the ABI may be associated with a tumour-related damage at the level of the cochlear nucleus [13]–[15]. Thus, for the stimulation at a higher level within the central auditory pathway proximal to the damaged cochlear nucleus, the inferior colliculus (IC) was chosen as target for a novel auditory prosthesis assigned as auditory midbrain implant (AMI; for review see [15], [16]).

As a result of insertion injury and foreign body reaction, fibrosis and gliosis occur. Neurons and neuropil decrease around the implantation site in the midbrain [17], [18], whereas the glial cell density is up-regulated up to 500 µm away from the array. This results in a fibrillary sheath formation of approximately 50 µm thickness [19]. Gliosis around a neuroprosthetic stimulation electrode [17], [19] increases the distance of the electrode to the target structure and by that the response thresholds. Thus, a focused activation of neurons is hindered.

One measure to enhance the clinical outcome of the patients receiving prostheses for neurostimulation may be the improvement of the neuron-electrode interaction by modifying the (surface) attributes of the implant as has been demonstrated recently for CI [20]–[23].

The IC acts as a major converging centre for ascending and descending auditory information (for review see [24]). In addition, there are also strong connections with non-auditory structures such as the superior colliculus, substantia nigra, and the somatosensory cortex (reviewed in [25]). Hitherto, artificial activation of the IC has been used for patients with retrocochlear auditory disorders. As a target of neuroprosthetic devices, an in vitro screening system for investigations of neuron-electrode interactions in the IC has not been reported yet.

So far, only organotypic cultures were established for the gerbil IC [26]–[28] and patch clamp methods on IC slices of mice and gerbil were published [29], [30]. Unfortunately, organotypic cultures are not suitable for examinations concerning the nerve-electrode interaction. Thus, the aim of this study was to establish an in vitro test system similar to the rat spiral ganglion neuron (SGN) culture for the inner ear: a dissociated culture of the rat IC.

## Materials and Methods

### 1. Overview of the experimental set up

For dissociation of inferior colliculi (IC), different experimental assays have been investigated as described in Materials and Methods (section chapter 3: Experimental assays and cell culture parameters investigated for the establishment of a dissociated IC cells culture) in detail. To allow for an overview, all experiments alongside with the major results will be shortly introduced below. In Table 1–2, a synoptic representation of all dissociation experiments is given.

**Table 1:** Synoptic representation of dissociation kits.

Assay	Kit/Method	Cell number [cells/well]	Cultivation period [d]
**Medium**	NTDK T$ *NTDK P$ *NTDK PN$ *SGN-protocol (0.1% trypsin)$ *	2×10^4$^ *	2 & 5
**Seeding density**	NTDK P*NTDK PN*SGN-protocol*	1×10^4^ *2×10^4^ *3×10^4^ *4×10^4^ *	2 & 5

*NTDK = Neural Tissue Dissociation Kit (from Miltenyi Biotech);*

*Each assay includes at least two independent preparations with 2–10 wells;*

$ *= supplemented Panserin 401;*

* *= supplemented MACS® Neuro Medium.*

**Table 2:** Synoptic representation of the proteolytic enzymes used for dissociation.

Enzymes	DNase I	Cultivation period [d]
0.125% trypsin (15′)	w/w/o	6
20 U papain (30′)	w/w/o	6
20 U papain (90′)	w/w/o	6

*Each assay includes at least two independent preparations with 2–10 wells;*

*w/ = with DNase I.*

*w/o = without DNase I.*

Initial screening experiments were performed utilizing four dissociation methods (Neural Tissue Dissociation Kit [NTDK] T, NTDK P; NTDK PN, and SGN-protocol; cf. Materials and Methods, 3.1–3.3), two culture media (Panserin 401 and MACS® Neuro Medium; cf. Materials and Methods, 3.4), and different cell seeding numbers (1×10^4^, 2×10^4^, 3×10^4^, and 4×10^4^ cells/well; cf. Materials and Methods, 3.5). Based on these initial screening results, maintenance of neurons in MACS® Neuro Medium was improved when compared to Panserin 401. An optimal seeding density that still allows cell investigation was identified as 2×10^4^ cells/well.

Thereafter, two different protocols with fresh prepared solutions containing only the proteolytic enzymes papain or trypsin were investigated (cf. Materials and Methods, 3.6) utilizing the optimal medium and seeding density.

### 2. Common methods used for all experimental assays

#### 2.1 Animals and Preparation

The IC were dissected from neonatal Sprague-Dawley rats of both sexes (postnatal day 3–5). Animals were rapidly decapitated. This procedure was reported to and approved by the Laboratory Animal Science Centre of the Hannover Medical School. The study was conducted in accordance with the German ‘Law on Protecting Animals’ (§ 4/03 TierSchG) and with the European Communities Council Directive 86/609/EEC for the protection of animals used for experimental purposes.

**Figure 1:**
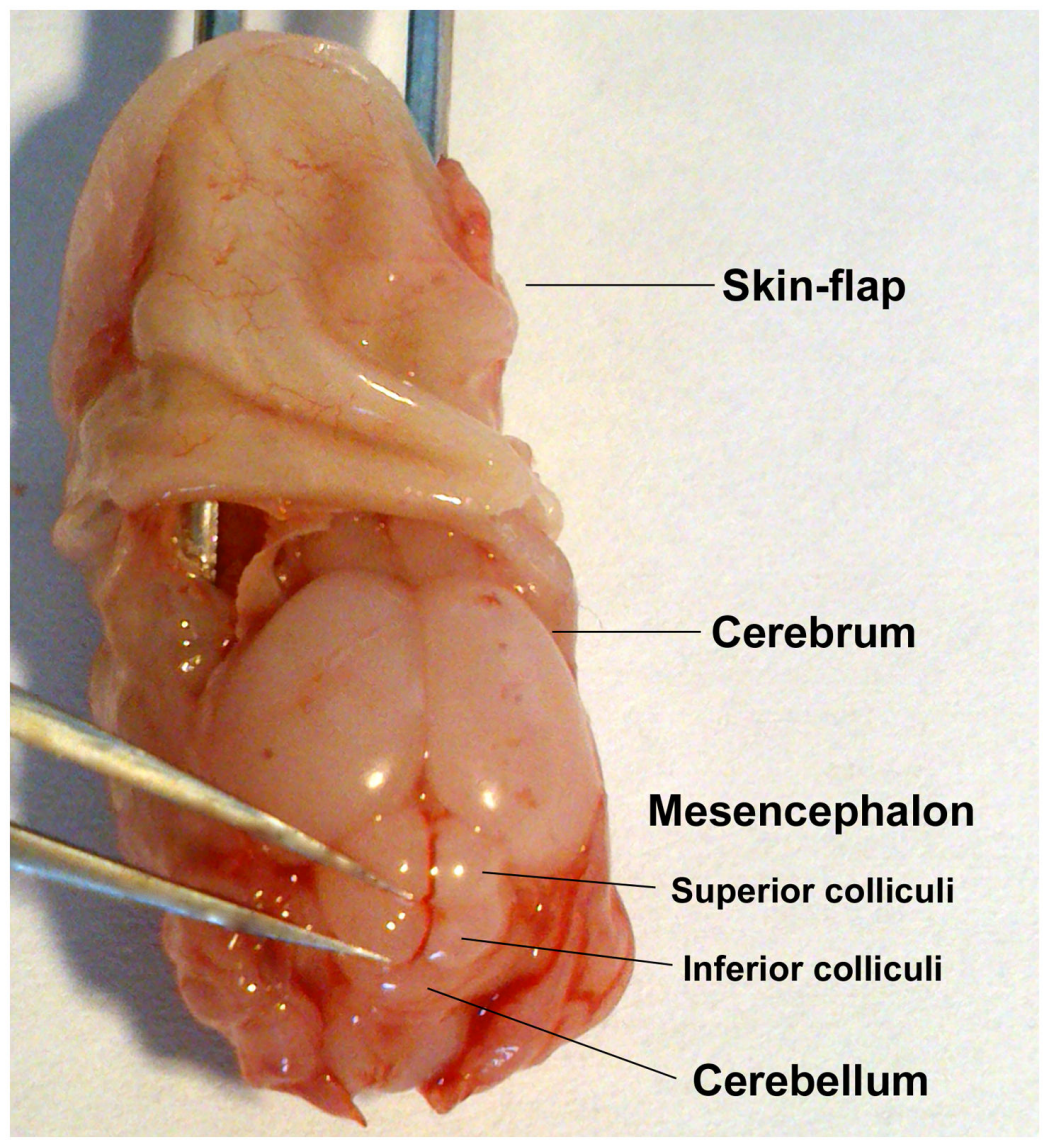
Anatomical overview for extraction of IC. With forceps fixed head of a neonatal rat (P3). After flapping the skin rostrally (top) and removing the calvaria, the following overview is given (from rostral to caudal): forebrain, midbrain, and hindbrain. The midbrain consists of the corpora quadrigemina, which are composed of the two rostral superior colliculi and the two caudal inferior colliculi (IC). The IC were elevated utilizing a Dumont No. 5 forceps (left).

After decapitation, the skin was removed from the occiput to the rostrum. To detach the skull, three incisions were enforced: cutting the os nasale (nasal bone) accounted for the first incision. The second and third incisions were performed bilateral from the angulus oculi lateralis to the lateral margin of the foramen magnum by preserving the temporal bone. Then, the calvaria could be removed carefully with forceps and an overview was given upon the forebrain (cerebrum, prosencephalon), midbrain (mesencephalon; especially the tectum: corpora quadrigemina), and the hindbrain (cerebellum, rhombencephalon) (Fig. 1). To extract the IC, the midline of the caudal two corpora quadrigemina (inferior colliculi) was cut and elevated using a Dumont No. 5 forceps (Fig. 1). Thereafter, both sides of the IC were carefully separated from each other avoiding extraction of the underlying tegmental brainstem (e.g. pedunculi cerebri). The isolated IC were kept either in ice-cold Ca^2+^/Mg^2+^-free Hank's Balanced Salt Solution (HBSS, gibco®, Invitrogen, Karlsruhe, Germany) or bovine serum solution for further processing (cf. Materials and Methods, 3.). This solution consists of 0.5% bovine serum albumin (BSA; Sigma Aldrich, Taufkirchen, Germany) in phosphate buffered saline (PBS; PBS tablets without Ca^2+^, Mg^2+^, and phenol red, gibco®) and is denoted as PBS/BSA.

The Eppendorf vials containing the storing solution were weighted before and after the addition of the tissue to determine the weight of the extracted IC in mg. Cell numbers obtained after dissociation were related to the weight of the IC tissue.

#### 2.2 Coating of cell culture wells

For improved cell adhesion, 96-well plates (NUNC, Thermo Fisher Scientific, Langenselbold, Germany) were coated with 100 µl poly-D/L-ornithine (0.1 mg/ml; Sigma Aldrich) and laminin (0.01 mg/ml; natural mouse laminin, Invitrogen) as described previously [31]. Wells were washed and prefilled with 50 µl of culture medium (for medium composition cf. Materials and Methods, 3.4) prior to the addition of the suspension containing the dissociated cells.

#### 2.3 Tissue dissociation and cultivation

The tissues were digested using different dissociation kits and protocols (cf. Materials and Methods, 3.) as well as proteolytic enzymes to obtain a homogenous cell suspension. After enzymatic and mechanical dissociation, the cells were counted in a Neubauer cytometer using the trypan blue (1%; Sigma Aldrich) exclusion test followed by seeding at different densities (cf. Materials and Methods, 3.; Table 1–2).

Cultivation of the cell suspensions was performed for 2–6 days in a humidified incubator at 37°C and 5% CO_2_ (cf. Materials and Methods, 3.; Table 1–2). Media were exchanged every three days. All samples were fixed with 4% paraformaldehyde (PFA; Merck Millipore, Darmstadt, Germany) diluted in PBS for 10 min at room temperature (RT). After fixation, cells were washed three times with PBS for 10 min each.

#### 2.4 Immunocytochemistry of dissociated IC cultures

For characterization of the cell types and their morphology as well as the evaluation of the neuronal yield and neuronal outgrowth, cultures of each experimental setting were stained immunocytochemically: PBS was removed from the cells and they were permeabilized with 0.5% Triton X-100 (Sigma-Aldrich) in PBS (PBT) for 3 min at RT. Then, cells were washed three times with PBS (for 3 min). Samples were blocked for 1 h at RT with blocking buffer containing 5% FCS. They were washed three times with PBS (3 min) followed by incubation for 1 h with primary antibodies diluted in antibody dilution buffer containing 2% FCS and 1% BSA in PBS. Afterwards, cells were washed again three times with PBS (5 min). Secondary antibodies were also diluted in antibody dilution buffer and incubated for 1 h at RT. Finally, cells were repeatedly washed three times with PBS (3 min) and stored in PBS containing 1% penicillin/streptomycin (Biochrom) at 4°C.

All primary and secondary antibodies are listed in Table 3–4. Since secondary antibodies are conjugated with fluorophores, incubation was performed in darkness to avoid photobleaching. Negative controls were performed by omitting the respective primary antibody from the protocol.

**Table 3:** Primary antibodies.

Primary antibody	Dilution	Company & Cat.-No.
***Identification of neurons***		
Monoclonal mouse anti-neuronal class III β-tubulin antibody (TUJ1)	1∶250	Covance #MMS-435P
Monoclonal mouse anti-Neurofilament 200 kD-antibody (NF)	1∶250	Novocastra #NCL-NF200
Polyclonal rabbit anti-growth associated protein 43 antibody (GAP-43)	1∶500	Abcam #ab16053
***Further characterization of cell subtypes***		
Polyclonal rabbit anti-glial fibrillary acidic protein antibody (GFAP)	1∶1000	Abcam #ab7779
Mouse monoclonal anti-MAG antibody (MAG)	1∶400	Abcam #ab89780
Polyclonal rabbit anti-S100 antibody	1∶100	Sigma Aldrich #S2644

**Table 4:** Secondary antibodies.

Secondary antibody	Dilution	Company & Cat.-No.
Goat anti-mouse IgG (H+L) Alexa Fluor®488	1∶400	Jackson ImmunoResearch *#115-545-003*
Goat anti-mouse IgG (H+L) Alexa Fluor®488	1∶400	Jackson ImmunoResearch *#115-545-003*
Goat anti-rabbit IgG (H+L) Alexa Fluor®594	1∶400	Jackson ImmunoResearch *#111-585-144*
Goat anti-chicken IgY (H+L) Cy™3	1∶400	Jackson ImmunoResearch *#103-165-155*

Neurons were imaged using an inverted microscope either from Olympus (CKX41) coupled with a CCD-camera (Colorview XS, SIS, Olympus, Münster, Germany) and were analysed using Cell∧D (SIS; Olympus) or from Zeiss (Jena, Germany; Axio Observer) with a HV-D30 camera (Hitachi Kokusai Electric Inc., Tokyo, Japan) together with the imaging software Axio Vision (Zeiss).

#### 2.5 Microscopical evaluation of the cultures

Cell cultures were observed under transmitted light microscopy (Olympus CKX41; Hamburg, Germany) after cell seeding, before and after aspiration and replacement of media as well as at the end of the experiment before and after fixation. After fixation, assays were rated from one consistent observer under transmitted light and after immunolabeling with epifluorescence. Following criteria were used to assess the efficiency of the various dissociation protocols, parameters, and culture conditions:

- Confluency (i.e. the coverage of well by the cells; estimated in %)
- The presence of cell clusters
- Neuronal yield
- Cytoarchitecture
- The presence of cell debris/purity of dissociation

#### 2.6 Atomic force microscopy of IC cultures

After dissociation with papain (30 minutes, cf. 3.6), 1×10^4^ cells were seeded in laminin-ornithine coated Petri-dishes (TTP, Trasadingen, Switzerland) and cultured for 7 days. To investigate cell morphology, cultures were fixed with 4% PFA and the Petri-dishes were mounted on an Axio Observer D1 inverted microscope (Zeiss, Jena, Germany) by means of a Petri-dish heater (JPK-Instruments AG, Berlin, Germany). After a cell was selected using light microscopy, the atomic force microscope (AFM, JPK-Instruments AG) was mounted on the top of the inverted microscope and the maximum scan size of piezo actuators on x-y-plane (100×100 µm) was selected to cover as much of single cell bodies as possible. All topography investigations were performed in contact mode in PBS using cantilevers characterized by extra-long tips and low nominal spring constants of 0.32 N/m (Biotool XXL, Nanotools, München, Germany) and 0.2 N/m (CSC21/AlBS, Mikromasch, Tallinn, Estonia). Best results were obtained by using setpoints of max. 1 nN and line rates of 0.08–0.15 Hz with pixel values of 512×512 over scan fields of 100×100 µm. To optimize the visualization of cells, AFM images were finally processed by means of a data processing software (JPK-Instruments AG, v.4.2.50). For measuring the actual heights of the investigated cells in a certain region by means of the data processing software, a cross section line was drawn along the desired cell region in the AFM height-measured image.

### 3. Experimental assays and cell culture parameters investigated for the establishment of a dissociated IC cells culture

In the first screening, four different dissociation protocols and two different culture media were tested. We used three kits from Miltenyi Biotech (Bergisch Gladbach, Germany): NTDK T (‘T’ stands for trypsin), NTDK P (‘P’ corresponds for Papain), and NTDK PN (‘PN’ stands for postnatal neurons and the enzymatic composition is unknown to the customer). Additionally, an established dissociation protocol for SGN was included as reference in the test series denoted as ‘SGN-protocol’.

In a second screening, we determined a suitable seeding number (cf. Materials and Methods, 3.5). Dissociated cells were seeded at a density of 2×10^4^ cells/well and cultivated for 48 hours or 5 days. A detailed description of the protocols and the culture media used is given below.

For the final experiments, we evaluated the proteolytic enzymes papain and trypsin (cf. Materials and Methods, 3.6).

#### 3.1 Dissociation of the tissue with NTDK T and P

The tissues were stored in HBSS and slightly centrifuged for a few seconds. The supernatants were discarded, 1 ml fresh HBSS was added and the samples were transferred for enzymatic digestion to a Falcon tube (Sarstedt, Nümbrecht, Germany) containing pre-warmed enzyme mix 1 (975 µl). After incubation for 15 min at 37°C, 15 µl of enzyme mix 2 was added. The samples were mechanically dissociated (triturated) with a 1000 µl pipette tip followed by incubation for 10 min at 37°C and further trituration with a 1000 µl and a 200 µl pipette tip for complete tissue homogenisation. After a third incubation for 10 min at 37°C, 20% fetal calf serum (FCS; Biochrom, Berlin, Germany) was added, the cell suspension was applied to a 70 µm pre-separation filter (Miltenyi Biotech) placed on a new Falcon tube and replenished with HBSS to the final volume of 10 ml. The sample was centrifuged at 800 rpm for 10 min at RT. The supernatant was discarded. The pellet was washed once with 10 ml HBSS followed by centrifugation (800 rpm, 10 min, RT). The second washing was performed with 10 ml unsupplemented culture medium followed by centrifugation and resuspension of the cells in culture medium (cf. Materials and Methods, 3.4).

#### 3.2 Dissociation of the tissue with NTDK PN

Prepared IC were stored in sterile filtered PBS/0.5% BSA until dissociation. All used materials were rinsed briefly with PBS/BSA. After sedimentation of the tissue, the supernatant was discarded and the sample was washed with 1 ml fresh PBS/BSA. Pre-warmed enzyme mix 1 (980 µl) was added for suspension of the cell pellet and incubated at 37°C for 15 min by gentle inversion of the tube every 5 min. After adding 15 µl of enzyme mix 2, samples were triturated carefully with a (pre-rinsed) 5 ml pipette (Sarstedt) ten times to avoid air bubbles followed by incubation for 10 min at 37°C. In addition, 7 µl of enzyme mix 2 were added and the tissue was further triturated using a 2 ml (ten times) and a 1 ml (35 times) pipette tip. The cell suspension was applied to a 70 µm pre-separation filter placed on a 15 ml Falcon tube and was replenished with PBS/BSA to a final volume of 10 ml. The mechanical pre-dissociated sample was centrifuged (800 rpm for 10 min, RT) and the supernatant was discarded. The cell pellet was resuspended with 1 ml PBS/BSA and triturated again with a 1000 µl and a 200 µl pipette tip for complete mechanical dissociation.

After determination of the total cell yield (cells/ml), the volume of the suspension (in PBS/BSA) was adjusted with media to the desired seeding number. After allowing the cells to adhere, the medium was replaced after one day of seeding and thereafter every third day.

#### 3.3 SGN-protocol

In HBSS stored IC were dissociated according to the SGN-protocol. The enzymatic digestion was performed for 20 min at 37°C with 0.1% trypsin (Biochrom) and 0.01% ( = 200 Units/ml) DNase I (#11284932001, Roche, Mannheim, Germany) diluted in HBSS as described previously [32]. To stop the enzymatic digestion, FCS (10%) was added to the vials. Samples were washed four times with medium prior to the mechanical dissociation that was performed by trituration using filter tips of different sizes. For details according to the mechanical dissociation the reader is referred to a recent publication [33]. In addition, samples were filtered with a 70 µm pre-separation filter (Miltenyi Biotech).

#### 3.4 Culture media

Two different culture media were tested for all conditions of the first screening experiments (NTDK T, P, PN, and SGN-method). The first medium consisted of Panserin 401 (PAN Biotech, Passau, Germany) and was complemented with N2 supplement (1 µl/ml; Invitrogen), insulin (4 mg/ml, Sigma Aldrich), and penicillin (300 Units/ml; penicillin G, Biochrom). Additionally, 4-(2-hydroxyethyl)-1-piperazineethanesulfonic acid (HEPES; 1 M; Invitrogen) was used in a final concentration of 23.4 mM and glucose (40%, Braun, Melsungen, Germany) was diluted with PBS to a final concentration of 6 mg/ml. This medium has been successfully used for the cultivation of SGN [33].

The second medium used was MACS® Neuro Medium (Miltenyi Biotech) supplemented with penicillin G (300 Units/ml, Biochrom), 2 mM L-Glutamine (from a 10 ml stock of 200 mM sterile filtered solution; 0.29228 g/10 ml PBS, Sigma Aldrich), and 1× B27 (MACS® Supplement B27 Plus, 50×, Miltenyi Biotech).

This supplemented MACS® Neuro Medium was used also for all experiments hereafter.

#### 3.5 Determination of a suitable seeding number

Within this seconds screening, the two (NTDK P and PN) out of three kits with the best results and the SGN-protocol were used to test four different cell seeding numbers (i.e. 1×10^4^, 2×10^4^, 3×10^4^, and 4×10^4^ cells/well; cf. Table 1). Cells were fixed with PFA after 48 hours or 5 days of cultivation in a humidified atmosphere.

#### 3.6 Evaluation of the proteolytic enzymes for cell dissociation

Fresh prepared solutions containing the key proteolytic enzymes of the kits were compared in MACS® Neuro Medium and the ideal seeding density established in the screening experiments. For this purpose, 0.125% trypsin/EDTA (Biochrom) and 20 U papain (Papain Vail PDS Kit, Worthington Biochemical Corporation, Lakewood, USA) were tested in combination with DNase I according to Tabata and colleagues [34]. After sedimentation of the samples stored in HBSS, the supernatants were discarded and the samples were washed with 1 ml fresh HBSS, followed by removing of the supernatants. Either 0.125% trypsin or 20 U papain (both diluted in 1 ml HBSS, respectively) were added to the tissues and incubated for 15 min (trypsin), 30 min and 90 min (papain), respectively, at 37°C. The digestion was stopped with 10% FCS and samples were washed twice with HBSS. The trituration of the digested IC was performed either in 1 ml HBSS/DNase I (5000 U/ml; Roche, Mannheim, Germany) or in HBSS without DNase I by using 1000 µl and 200 µl pipette tips. After centrifugation (1000 rpm, 5 min, RT), supernatants were removed carefully. The cells were resuspended with supplemented MACS® Neuro Medium, seeded at a density of 2×10^4^ cells/well and cultivated for 6 days.

## Results

### 1. Summary of the results of the screening experiments

#### 1.1 Improved neuronal yield by the use of MACS® Neuro Medium

After dissociation with the four different digestion protocols, the following cell yield (c) was obtained per mg tissue (c×10^4^/mg; mean ± SD): NDTK T = 1.25±0.01, NTDK P = 1.98±0.36, NTDK PN = 1.30±0.15, and SGN-protocol = 1.50±0.25.

**Figure 2:**
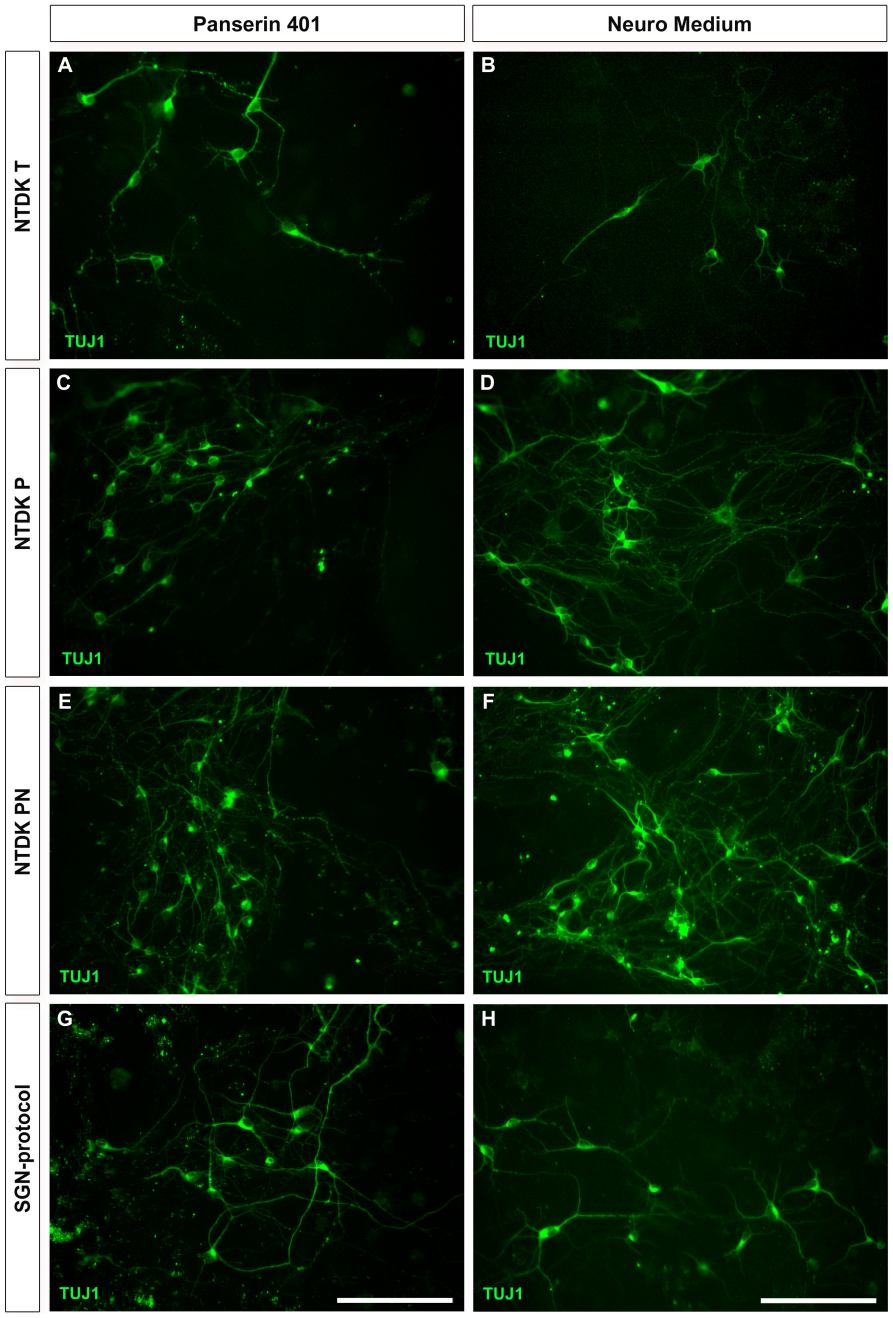
Immunocytochemical results of different dissociation protocols and media. Improved neuronal yield and branching was observed in cells cultivated for 5 days with MACS® Neuro Medium (right column). By contrast, cells cultivated with Panserin 401 (left column) showed less branching with prominent neurite fragmentation (A, E). IC tissue was dissociated with different protocols: NTDK T (A, B), NTDK P (C, D) and NTDK PN (E, F) as well as SGN-protocol (G, H). After fixation and labelling with TUJ1 (green), poor neuronal yield was obtained after dissociation with NTDK T independent from the cultivation medium. Scale bar: 100 µm.

Cultivation of dissociated cells (from all protocols) in supplemented MACS® Neuro Medium (Table S1a) resulted in an increased survival of cells evaluated 2 and 5 days after seeding. When compared to Panserin 401 (Table S1b), confluency was improved by approximately 10% in cultures maintained in MACS® Neuro Medium. After labelling with anti-neuronal class III β-tubulin (TUJ1) antibody, the neuronal yield was increased in cultures incubated with MACS® Neuro Medium (cf. Figure 2, left column). In addition, neurons developed branching neurites forming a neuronal network 5 days after seeding, whereas neurons in Panserin 401 showed fragmented neurites varying in length and branching. This fragmentation was consistent within all assays. Thus, supplemented Panserin 401 was not able to promote sufficient neuronal support for longer cultivation periods and was excluded for further experiments. Supplemented MACS® Neuro Medium was used for all following experiments as standard culture medium.

The highest cell yield per mg tissue was obtained with the NTDK P dissociation protocol. Distribution of neuronal cells, neurite length, and the generation of a neuronal network was evaluated after labelling with TUJ1 antibody. Processing of IC tissue with NTDK P, NTDK PN, and SGN-protocol resulted in an enhanced neuronal yield, regular distribution of cells and development of neuronal networks (Fig. 2C–H).

Double-staining of TUJ1 in combination with growth associated protein 43 (GAP-43; identification of growth cones in neurons) showed that most TUJ1 positive cells were co-stained with GAP-43. Both antibodies stained somata as well as neurites. However, single, intense, and dot-shaped structures could be identified by GAP-43 staining in cells that neither exhibited neuronal morphology nor were labelled with the TUJ1 antibody (Fig. S1, left column). Anti-neurofilament 200 kD (NF) antibody - a second neuronal cytoskeletal marker - was also tested and resulted in a staining of neurons comparable to that of TUJ1 but poor in intensity (data not shown). Thus, only TUJ1 was utilized as standard marker for neurons in all following investigations.

Distribution of astrocytes and the discrimination from neurons was judged after labelling with antibodies against the glial fibrillary acidic protein (GFAP; a class-III intermediate filament labelling astrocytes) and TUJ1, respectively. An intense cytoplasmic immunolabeling of the cytoskeletal protein GFAP was evident in cells with astrocytic phenotype. Immunocytochemistry and the astral-like cytoarchitecture of those cells supported their astroglial identity as described previously for primary astrocytes [35], [36]. This was corroborated after co-staining of the cultures with TUJ1 and GFAP, where the neuronal-specific labelling was absent in GFAP positive cells. Neurons were most likely surrounded and probably connected with GFAP positive cells which indicate the physiological function of astrocytes. This phenomenon was quite regular only when NTDK P, NTDK PN, and the SGN-protocol were used as dissociation procedure (Fig. S1, right column).

Dissociation of the IC with the NDTK T resulted in the lowest cell numbers per mg tissue (1.25±0.01). In addition, the digestion with the NTDK T resulted in low confluences of approximately 5–10% (cf. Table S1a–b). After staining, especially the neuronal yield was low (Fig. 2A, B). Glial cells present usually thick, astral-like processes. However, when dissociated with the NTDK T, they tend to develop only few, less branched processes and only partly surrounded the neurons (Fig. S1, left column). Altogether, the neuronal yield gained by the NTDK T was quite poor, co-localization of neurons and cells with proliferative capacity (like glial cells) do not grow under extended cultivation period (Table S1a). Thus, this method was excluded for further experiments.

#### 1.2 Increase of seeding density did not improve culture conditions due to an increased formation of cell clusters and cell debris

Different seeding numbers were investigated in order to identify the optimal seeding density for improved cell distribution, proliferation, and survival after seeding (especially neuronal survival). Thus, four different seeding numbers (i.e. 1, 2, 3, and 4×10^4^ cells/well) were tested (Table 1) in two independent preparations. The following cell yield (c×10^4^/mg) was obtained after digestion with three different dissociation protocols: NTDK P = 1.65±0.09, NTDK PN = 1.13±0.04, and SGN-protocol = 1.85±0.58.

Seeding of the cells with a density of 1×10^4^ cells/well resulted in a minor coverage of the well even after 5 days of cultivation. The increase of the seeding density led to a homogenous distribution of the cells after 5 days of cultivation (Table 5). However, at higher seeding densities, cell cluster and cell debris started to appear (from 2×10^4^ cells/well onwards) and accumulated (3×10^4^ cells/well) especially, when the SGN-protocol was used (for details see below, Table 6). Already at low seeding densities, cultures dissociated with the SGN-protocol showed the formation of cell clusters and the presence of cell debris (Fig. S2, left column). In general, these clusters were anchored and located above the cell layer and hampered the optical evaluation. Thus, rating of cytoarchitecture was not possible even after medium seeding density of 2×10^4^ cells/well and 5 days of cultivation (data not shown). The debris and the cluster could not be reduced in the dissociation process by applying the cell suspension to a cell strainer or in the well by washing out with exchange of the media. The increase of the seeding density could only improve the neuronal yield (for cells dissociated according to the NTDK PN and the SGN-protocol) until 2×10^4^ cells/well. By further increase of the seeding number, neuronal yield, neurons with intact cytoarchitecture and neuronal network formation were reduced (Fig. S2, middle and right column). Thus, we determined 2×10^4^ cells/well as the most suitable seeding density.

**Table 5:** Results$ obtained from the screening experiments for seeding densities.

	SD [cells/well]	Confluency [%]	Formation of cell clusters	Cell debris	Neuronal yield
**NTDK P**	1×10^4^	20	(+)	−	+§
	2×10^4^	20–30	(+)	−	(+)§
	3×10^4^	30–40	+	+	+
	4×10^4^	40	+	+	+
**NTDK PN**	1×10^4^	30–40	(+)	+	+§
	2×10^4^	40–50	(+)	(+)	+(+)
	3×10^4^	50–60	+	+(+)	++
	4×10^4^	70–80	+	(+)	+
**SGN**	1×10^4^	60–70	++	++	+§
	2×10^4^	50–60	++	++	+(+)§
	3×10^4^	50	++	++(+)	+(+)§
	4×10^4^	80	++(+)	+++	+(+)§

*CP: cultivation period;*

*SD: seeding density.*

$ *For a better overview, only results from one preparation were shown for 5 days of cultivation.*

§ *spheroidal morphology with no or short neurites (restricted neuronal morphology);*

*Confluency: rated confluency in per cent; optical evaluation of the whole well under bright-field conditions using a 40-fold magnification;*

*Cell debris, conglomerates, and neuronal yield.*

*−: no debris, conglomerates, or neurons;*

*+: poor amount; +(+): poor-middle;*

*++: middle amount; ++(+): middle-high;*

*+++: high amount.*

**Table 6:** Results obtained with the proteolytic enzymes.

MACS® Neuro Medium	0.125%Trypsin		20 U Papain 30 min		20 U Papain 90 min	
DNase I	without	with	without	with	without	with
**Confluency [%]**	40–60	40–80	40–75	40–70	20–40	<5
**Cell debris**	++	+	−	−	+	++
**Neuronal yield**	+(+)	++	++(+)	++	+	−

*No formation of cell clusters was observed.*

*Confluency: rated confluency in per cent; optical evaluation of the whole well under bright-field conditions using a 40-fold magnification;*

*Cell debris, conglomerates, and neuronal yield.*

*−: no debris, conglomerates, or neurons;*

*+: poor amount; +(+): poor-middle;*

*++: middle amount; ++(+): middle-high;*

*+++: high amount.*

#### 1.3 The degree of dissociation seemed not to be a key element neither for enhancing the distribution of cells nor the neuronal yield

The impact of the degree of dissociation on cell behaviour and survival was investigated using the NTDK P and NTDK PN (seeding density 2×10^4^ cells/well). Although an intact cytoarchitecture was observed and an adequate neuronal yield was obtained by both kits, no differences between a fine and a rough dissociation procedure were obvious (data not shown). Thence, the degree of dissociation seemed to have no influence upon the confluency of the cells and the neuronal yield.

#### 1.4 Prolonged cultivation period enhanced extension of neurites and the formation of a neuronal mesh

After 2 or 3 days of incubation, the well surface was covered (confluences between 10% and 70% with a mean of approximately 30%) and the distribution of cells under bright-field varied between growth in preferred zones (marginal areas as well as centre of the well) and a homogenous covering of the well. After 5 days of incubation, an increased covering of the surface of the well and a smaller amount of cell clusters was observed. Generally, cells were distributed homogenously (without any preferred growth zone).

Dissociated IC cells were labelled with TUJ1 individually or in combination with GFAP. Since both markers represent cytoskeletal marker, we were able to assess - besides the neuronal yield - the cytoarchitecture and their differences within the kits. In double-stained cultures, the ratio between neurons and astrocytes was estimated. In general, this ratio was in favour of glial cells due to their capacity to proliferate.

Stained neurons showed longer neurites. They were more branched or built a neuronal network. Nonetheless, fewer neurons with an intact cytoarchitecture were observed in the central areas of the well. The number of glial cells and branching characteristics were also augmented. This influence of the cultivation period was consistent for all experimental settings except for the NTDK T.

Thus, a suitable maintenance of dissociated IC cells with MACS® Neuro Medium and its supplements was achieved up to 7 days.

### 2. Results obtained with proteolytic enzymes

In the above screening experiments, MACS® Neuro Medium was evaluated as the appropriate medium for the maintenance of dissociated IC up to 5 days. For an ideal cell distribution and confluency that allows the evaluation of the cytoarchitecture, 2×10^4^ cells/well was determined as the best seeding density. From the dissociation protocols with altered parameters that were tested, best cell and neuronal yield was obtained by the use of the NTDK P and NTDK PN. The NTDK P contains papain as main proteolytic enzyme. In the following, the dissociation with fresh self-prepared digestion solutions containing only the enzymes of interest - trypsin (in another variation to the previously established SGN-protocol) and papain (the main component of the NTDK P) with and without the addition of DNase I during the trituration - were investigated.

For papain, two different incubation periods of the digestion (30 and 90 min) were tested. All cells were cultivated over a period of 6 days (Table 2). Two independent preparations were performed and the following cell yield (c×10^4^/mg) could be attained after dissociation: trypsin = 4.68±2.95, papain 30 min = 2.19±1.28, and 90 min = 2.37±0.21. Compared to the cell yield gained with the dissociation protocols used in the screening experiments, higher cell numbers were obtained with the fresh prepared solutions.

#### 2.1 Papain 30 min resulted in highly branched neurons and glial cells

**Figure 3:**
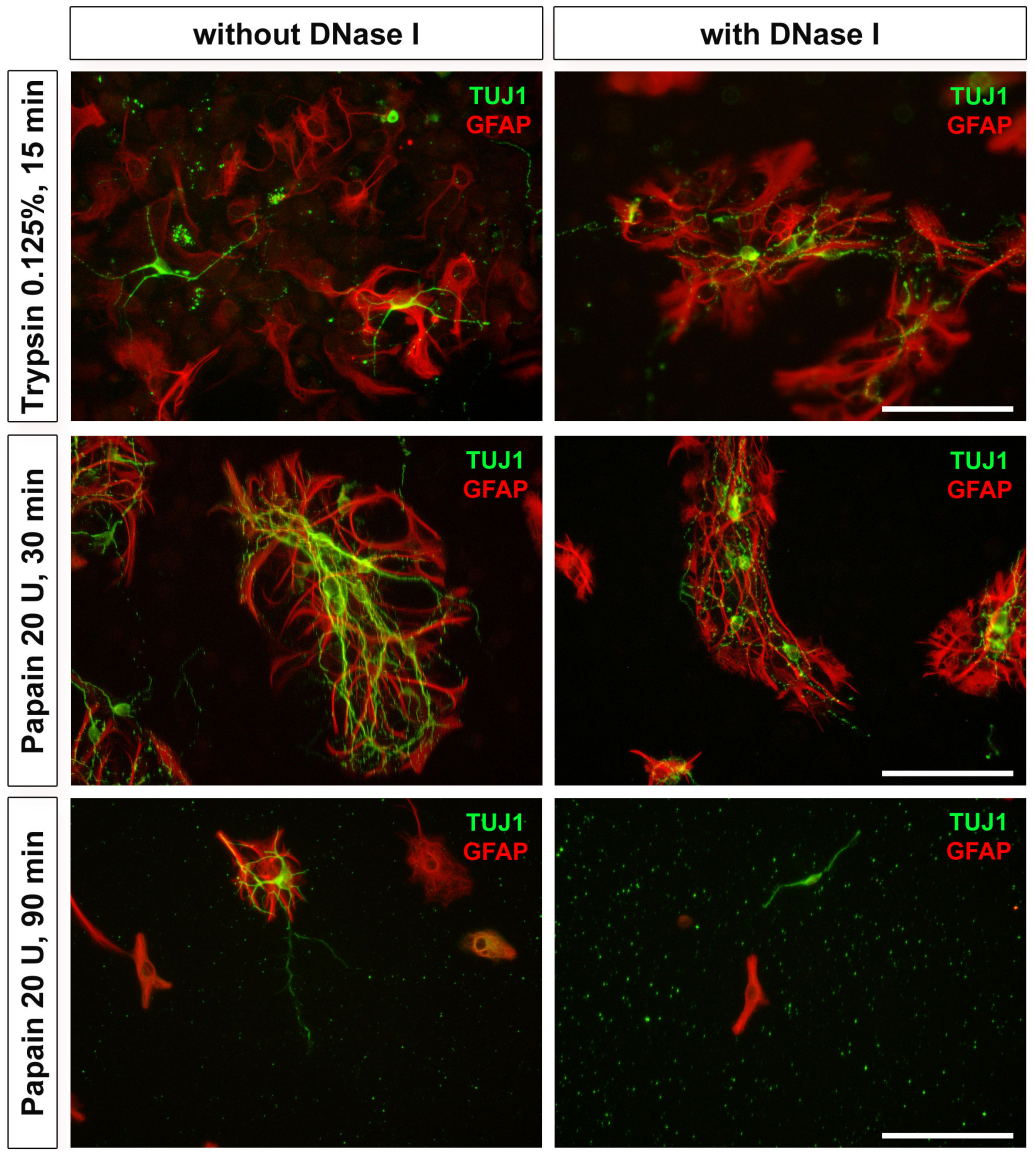
Fluorescent images from the testing of proteolytic enzymes. Cells digested either with trypsin (15 min: first row) or papain (30 min: second row and 90 min: third row) were triturated without (left column) and with DNase I (right column). Merged pictures of cells labelled with TUJ1 antibody (green) and GFAP antibody (red) were depicted. When comparing all depicted conditions, an improved cytoarchitecture with high neuronal (and glial) yield was obtained in cultures dissociated with papain for 30 min without DNase I. Scale bar: 100 µm.

After seeding, the rated (transmitted light) confluences for papain (30 min incubation) and trypsin were comparable (each varying between 40–60% and 40–75%, respectively; Table 6). However, differences in the yield of neurons and their branching prevalence were observed in favour of the papain dissociation. Furthermore, the neurons were scarcely present without building neuronal meshes when digested with trypsin. In addition, some debris was visible under fluorescence microscopy (Fig. 3, first row). By contrast, dissociated cultures generated with papain were free of any cell debris when incubated for 30 min (Fig. 3, middle row). However, extended dissociation time up to 90 min led to poor confluences (20–40%) and thus, also to a decreased survival of neurons. Glial cells were affected in their morphology (fewer and shorter branching). Under (red and green) fluorescence, a high amount of debris was visible in these wells (Fig. 3, third row). Thence, dissociation with papain for 30 min resulted in a higher yield of well distributed neurons, highly branched neurons building a neuronal mesh and well co-localized with GFAP positive cells (Fig. 3, middle row).

When adding DNase I for trituration, confluences under bright field evaluation of cultures after dissociation with papain and trypsin were comparable to those with HBSS trituration (Table 6). However, after cell-type specific staining (Fig. 3, right column), elongation of cells obtained from tissue triturated with DNase I was decreased compared to HBSS-triturated tissue (with the same enzymatic dissociation; Fig. 3, left column). If the digestion period with papain was increased up to 90 minutes, trituration with DNase I resulted in the lowest confluences (<5%) Survived neurons and glial cells showed abnormal morphology (deformed shrunken cell bodies with no or very short elongations) when compared to prolonged incubation with papain alone (Fig. 3, third row). Based on our results, addition of DNase I for trituration of cells seem not advantageous over the use of an enzyme-free trituration solution (HBSS). However, a wide range of DNase I concentration needs to be investigated in order to corroborate this result.

#### 2.2 Oligodendrocytes as a part of the dissociated IC culture

**Figure 4:**
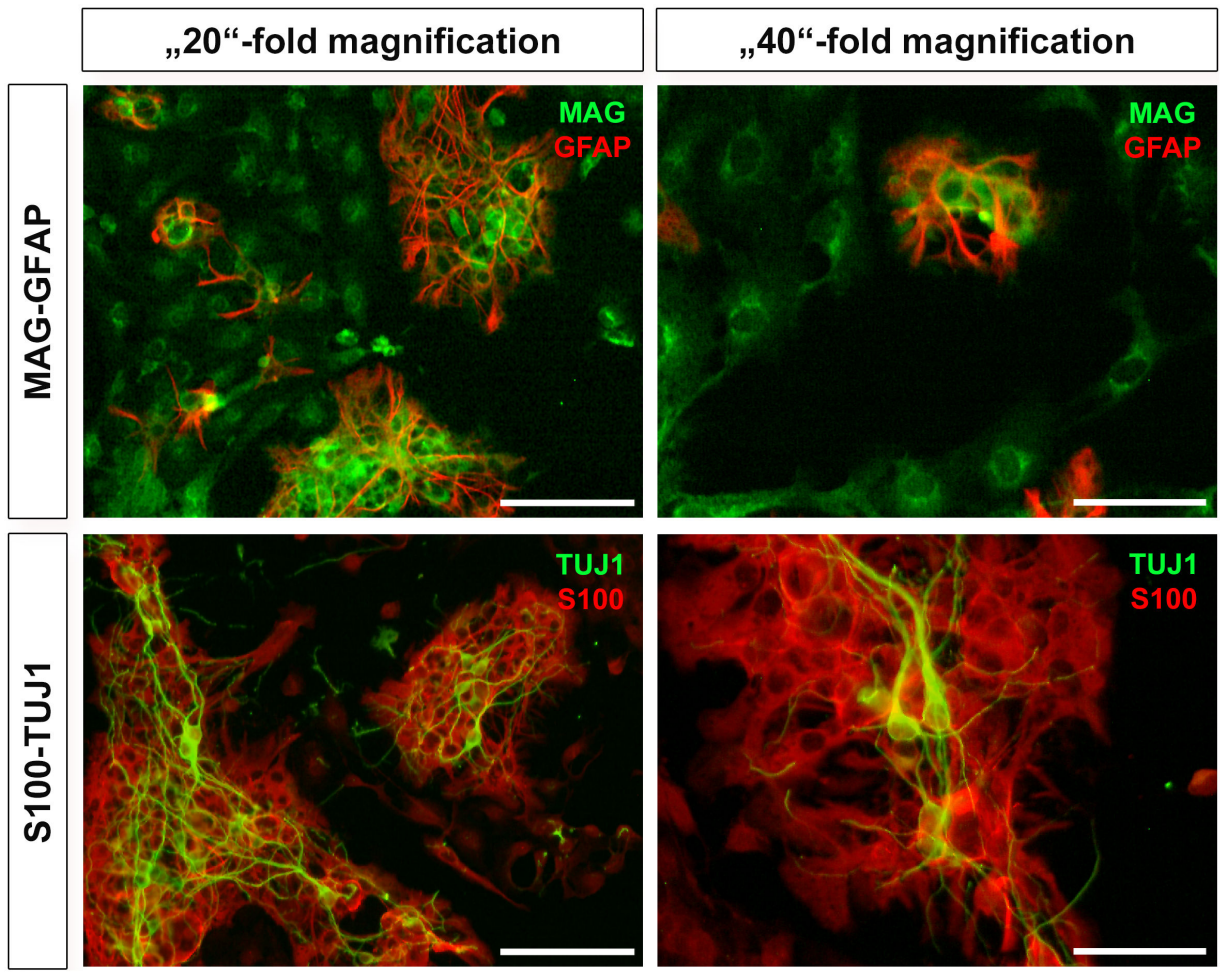
Fluorescence images of different antibodies for glial cell characterization. Papain digested cells were labelled with GFAP (red) and MAG (green) and are depicted in the first row. Positive staining for GFAP as well as MAG discriminate astrocytes as well as oligodendrocytes, respectively. A second astrocytic marker (S100, red) was also tested in combination with TUJ1 (green) and presented in the second row. Positive staining for S100 confirmed previously obtained results with GFAP (second row). Two different magnifications are presented in the left (20×; scale bar: 100 µm) and right column (40×; scale bar: 50 µm).

To discriminate glial cells (oligodendrocytes from astrocytes), cultures obtained after dissociation with papain (30 min, without DNase I) were stained with myelin-associated glycoprotein (MAG; adhesion molecule in postnatal neural development mediating sialic-acid dependent cell-cell interactions between neuronal and myelinating cells) and GFAP, respectively. MAG was also expressed in cells within the IC culture and showed an intense cytoplasmic immunolabeling localized around the nucleus. None of the cells were positive for both glial markers (GFAP and MAG; Fig. 4, first row), suggesting the oligodendrocytic phenotype of MAG positive cells.

Papain (30 min, without DNase I) dissociated cells were also stained with S100 antibody (calcium-binding protein synthesized in astrocytes and also other glial cells). Cells positive for S100 were located around TUJ1 positive cells within the IC culture. They revealed a cytoplasmic immunolabeling sparing the nucleus (Fig. 4, second row). Since this protein is not a cytoskeletal marker, the morphology of the S100 positive cells was not assessable. Nonetheless, their distribution around neurons was similar to those of the GFAP positive cells (astrocytes) indicating that immunolabeling with S100 referred to astroglia.

#### 2.3 AFM results

**Figure 5:**
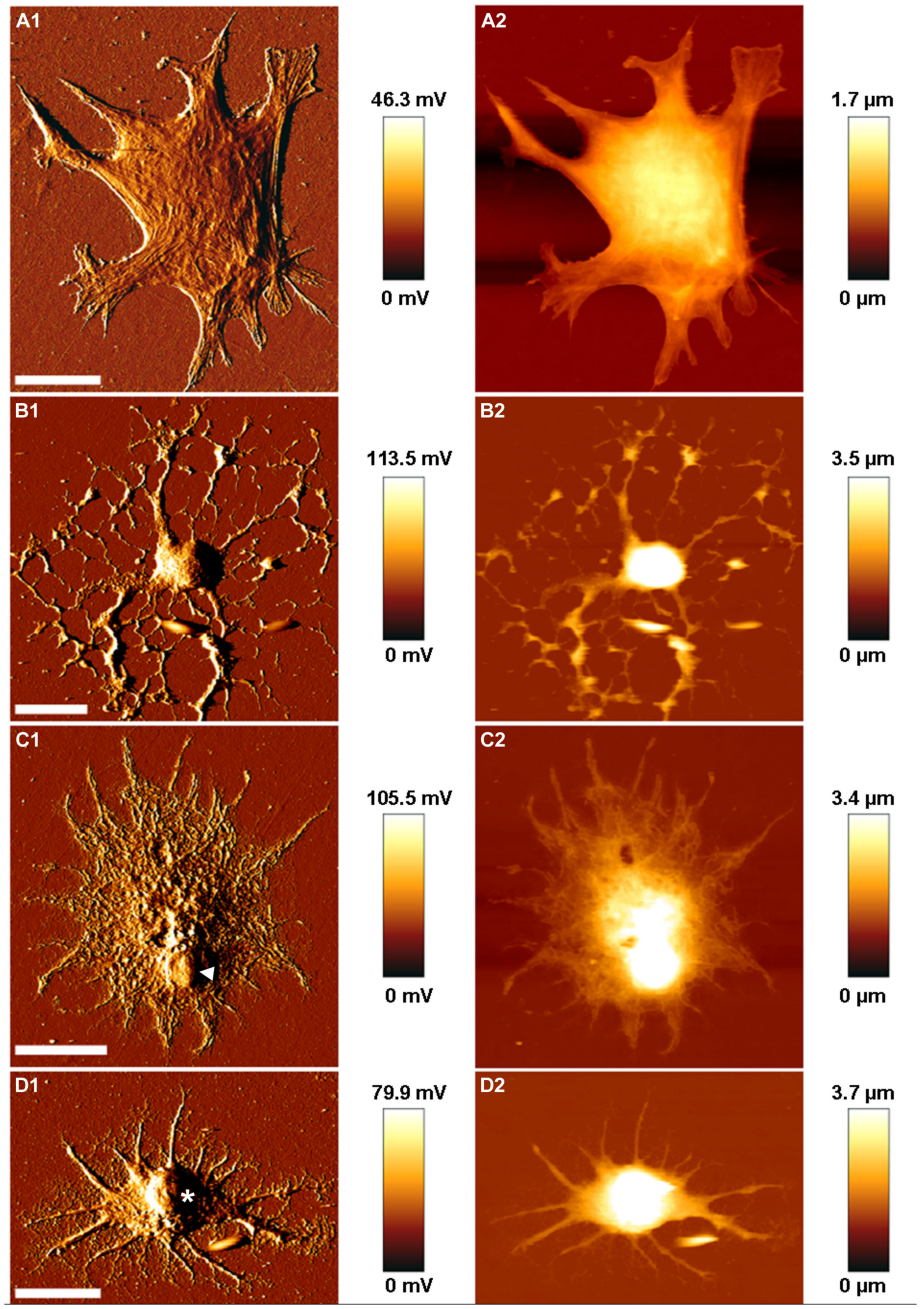
Representative AFM images of individual, dissociated IC cells. AFM error signal (1) and height (2) images of four morphologically different cells (A–D) are presented. For possible cell type specification, the reader is referred to Chapter 3.2, AFM results. Arrowhead and asterisk denote the nucleus. Scale bar: 20 µm.

Using AFM, the morphology of different cell types observed in the IC culture was investigated. Four morphologically different cell types, from which one certain cell is illustrated on Fig. 5, were revealed. Among the cells observed in the culture, some showed a flattened morphology, characteristic polygonal or irregular shape, and strong filaments within the soma (Fig. 5, A1–2). With a maximum height of about 1.5 µm in central cell region, this was the flattest cell measured within the culture. Within the brain, such a flattened morphology seems most likely for vascular cells. By contrast, the Fig. 5, B1–2 illustrates a cell with a less broad but higher (approx. 6 µm), round shaped soma. Numerous processes variable in thickness arouse from the soma and branched during elongation. Many of the smaller branched elongations seemed to be connected to each other. Within the Fig. 5, C1–2, a cell with a star-shaped phenotype was presented. With approx. 4 µm, the highest part of the cell was a dense, round structure (arrowhead) – probably the nucleus. Away from this dense area, the cell seemed to be loosen and formed a fine network tapering in many processes and the cell per se flattened out. The two cells depicted in Fig. 5, B1–2, and C1–2 row exhibit most likely glial cell morphology. The last cell depicted in Fig. 5, D1–2 showed a small soma with a prominent, dense, high (approx. 6 µm), and round structure indicating the nucleus (asterisk in Fig. 5, D1–2). In contrast to the second cell (Fig. 5, B1–2), various, thicker processes with many fine, bifurcated processes developed out of the soma, possibly presenting neurites.

### 3. Summary of the results

Reproducible results were obtained after digestion with the proteolytic enzymes trypsin and papain (30 min): sufficient in cell yield and distribution as well as less variations in confluency and neuronal yield. Cell clusters were absent. However, an excellent cytoarchitecture with oligodendrocytes as well as highly branched neurons and astroglia were only obtained after dissociation with papain for 30 minutes. Cell debris and the lowest survival were only visible within the cultures dissociated with papain for 90 minutes. Hence, a prolonged enzymatic digestion up to 90 minutes seems quite toxic for IC cells. Based on these results, digestion with papain for 30 minutes without DNase I for trituration was determined as the most suitable method for the dissociation of the IC tissue.

## Discussion

The developed protocol (dissociation of cells with papain for 30 min without DNase I trituration) provides reproducible results concerning sufficient cell and neuronal yield, distribution of cells, and confluency as well as a cytoarchitecture typical for healthy cells without the occurrence of cell clusters. We were able to cultivate the obtained cells for 2–7 days in complemented MACS® Neuro Medium. A neuromedium specifically designed to promote cultivation of cells of the central [37]–[41] and peripheral [42], [43] nervous system seems to be more suitable for the cultivation of the IC than the also tested Panserin 401.

All protocols published so far for papain use only one concentration (20 U/ml) but different incubation times (30–90 minutes, depending on the size of the specimens) and trituration procedures [34], [44]–[47]. Our results indicate that the size of the IC is too small for an extended incubation period (up to 90 minutes). Thus, papain presents the most gentle digestion enzyme for brain tissue, especially for small specimen size such as the IC. By contrast, the use of the kits for enzymatic digestion resulted in a tendency towards lower cell yield, which could be an indicator for additional ingredients that may affect the digestion and dissociation of the tissue. Since the concentration of the enzymes and the composition within the kits is unknown to the customer, we investigated self-prepared solutions containing the two main enzymes trypsin (0.125%) and papain. Previously, several groups reported that trypsin exerts higher toxicity upon central neurons than papain [34], [44]. Our results confirm this finding. Applied at a lower concentration (0.025%), a dissociated culture from the cochlear nucleus (CN) has been obtained after digestion with trypsin [48]. Thus, trypsin seemed to exert low to medium toxicity (depending on the concentration) on neurons.

Within the dissociated culture, most of the cell types of the IC were identified: neurons, astrocytes, and oligodendrocytes. In addition, besides neurons and glial cells, vascular cells can be found in the IC (and other structures of the central nervous system). Endothelial cells and pericytes (both vascular cells) form the blood-brain barrier together with astrocytes and several matrix molecules [49]. Using AFM, cells were judged according to their morphology: The flattened cell with a characteristic polygonal or irregular shape (Fig. 5, A1–2) could be addressed either to endothelial cells or pericytes [50], [51]. However, due to the strong filaments, this cell most likely is a pericyte [50]. The star-like phenotype of the cell presented in Fig. 5, C1–2 seemed to reveal astroglial identity, whereas the cell in Fig. 5, B1–2 could be addressed as an oligodendrocyte [52]. Although the neurites were relatively short even after 7 days of cultivation, due to the soma-nucleus the cell imaged in Fig. 5, D1–2 could be attributed to neurons. Alterations in the morphology could be caused by the reduced seeding density that was required for AFM measurements.

Since our focus was the establishment of a dissociated IC culture for investigations on neurons and glial cells, we verified these cell types immunocytochemically. The most sufficient neuronal yield and cytoarchitecture was obtained after digestion with papain. A slight fragmentation of neurites was observed in all tested conditions. One reason for susceptibility to fragmentation may the origin of the tissue: Purkinje neurons isolated from embryonic tissue showed an improved survival than those from neonatal animals [53], [54]. In addition, the fine branched neurites of primary embryonic Purkinje neurons tend to exhibit fragmentation after an extended cultivation period [53]. Neurite fragmentation may be also judged as a sign of apoptosis. Indeed, Tabata and colleagues [34] described that signs of apoptosis occur in Purkinje neurons isolated from early postnatal mice (P 4). Nonetheless, except for the fragmentation, we did not observe any other morphological signs of apoptosis, i.e. shrunken somata and cytoplasmic vacuoles [55], in our postnatal IC cultures.

Glial cells are divided into two families based on their germ layer origin: microglia (small size and mesodermal origin) and macroglia (large size and ectodermal origin). Macroglia can be further subdivided into oligodendrocytes and astrocytes with distinct functions. Oligodendrocytes are mainly responsible for the myelination process in the central and peripheral nervous system. After co-staining with anti-MAG (specific marker for oligodendrocytes) and anti-GFAP antibodies (only staining astrocytes), we were able to distinguish between oligodendrocytes and astrocytes. Thus, myelination in the IC starts at early postnatal stages as described previously by other groups based on stains of slices of the IC: at P 4 in the rat [56] and in gerbils [57] and between P 0 and P 7 in rats [58].

The maintenance of the extracellular environment and the stabilization of cell-cell communications within the central nervous system are warranted by astrocytes [35], [36], [59]. For example, primary embryonic Purkinje neurons died within a few days after omitting the astrocytic layer [53]. In histological slices of the IC of neonatal rats, Hafidi and Galifianakis [58] observed a different distribution pattern for GFAP and S100: at all stages, the cells of the IC were GFAP negative, whereas GFAP positive cells could be found in the surrounding areas of the IC and the whole brain. By contrast, S100 positive cells appeared at birth with a homogenous distribution across the whole IC. Interestingly, in our dissociated culture GFAP as well as S100 positive cells are present. The differences of the GFAP expression in vivo (histological slices) and in vitro can be explained by the influence of the seeding density on the yield of astrocytes and oligodendrocytes in dissociated cultures [60]. In addition, the microenvironment also determines the development of progenitor cells in astro- or oligodendrocytes [61]. Another cofounding factor could be the deepness of the manual dissection. Since the isolation of deeper tissue surrounding the IC cannot be excluded, we used triple labelling for TUJ1, GFAP, and S100 (Figure S3) to discriminate astrocytes (i.e. cells that were co-stained with anti-GFAP- and anti-S100-antibodies) from oligodendrocytes.

As described in the introduction, formation of gliosis after implantation of central neural prostheses may act as an isolator for electrical stimulation. Astrocytes represent the key component in reactive gliosis in adult CNS [62], [63]. In order to reduce gliosis, the influence of material properties especially on the adhesion and proliferation of glial cells is of interest. Thus, a dissociated culture reliably revealing all the different cell types is given with the herein presented protocol. It can be adjusted depending on the envisaged experiments: The preconditions for AFM measurements to investigate the impact of material properties can be realized by e.g. reducing the seeding number or cultivation period since single cells without neuronal networks and cell-cell-adhesions are requested [21]. On the other hand, if the goal is to screen novel materials or their modification in terms of biocompatibility, a homogenous culture with high cell yield is necessary and this can be realized using an increased seeding density and/or prolonged cultivation period.

Since the IC is connected with the superior colliculus, the substantia nigra as well as the somatosensory cortex (reviewed in [25]) and receives multisensory input (reviewed in [64]), a diverse population of neurons with possibly distinct functions may be expected within our dissociated culture. The majority of the neurons identified so far in the IC are GABA(γ-aminobutyric acid)-ergic [25] and account for inhibitory processes mediated via the IC [24], [64], [65]. The density of GABAergic neurons in the central nucleus IC varies between species: rats, the species we used in this study, contain more than 30% of GABAergic neurons, whereas for bats or cats, much lower densities are reported [25]. GABAergic input may alter dopamine neuronal activity since they synapse directly onto dopaminergic neurons in the ventral tegmental area [66]. In addition, non-GABAergic neurons have been observed within the IC: Based on recent findings, the presence of glycinergic [67] and glutamatergic neurons [68] as well as dopamine receptors [69] have been described. The GABAergic and glutamatergic system within the IC may influence the nigrostriatal and dopaminergic pathways. For example, electrical stimulation of the IC causes significant increase of extracellular dopamine levels in the frontal cortex of rats [70].

In order to enhance the performance of neuroprostheses for the auditory, motor and behavioral system, the herein presented protocol may be of relevance for the screening of material and surface properties on electrode-tissue interactions such as cytotoxicity, adhesion, proliferation, inflammation, intercellular interactions and neuroprotection.

## Supporting Information

Figure S1 **Immunocytochemical results for different dissociation protocols.** After dissociation with different protocols (NTDK T, NTDK P, NTDK PN and SGN-protocol), herein presented cells were cultivated with Neuro Medium and fixed after 5 days. Cells were labelled with TUJ1 (green) and GFAP (red). Scale bar: 100 µm.
(TIF)

Figure S2 **Fluorescence images of different seeding densities.** Best images from the cells dissociated with three different methods (NTDK P: left column; NTDK PN: middle column and SGN-protocol: right column) seeded at four different densities (from 1×104 cells/well: top row; to 4×104 cells/well: bottom row) are depicted. TUJ1 labelled (green) images were shown. Arrows indicate the formation of cell clusters and arrowheads single neurons. Scale bar: 100 µm.
(TIF)

Figure S3 **Triple staining of TUJ, GFAP, and S100.** Both, anti-GFAP and anti-S100 antibodies stain astrocytes. In the triple staining (merge), astrocytes stained for GFAP (red) and for S100 (blue) seemed to be identical and clearly distinct from neurons (green). Scale bar: 100 µm.
(TIF)

Table S1 (DOC)
