# Supplementary figures and images for: Dissociated Neurons and Glial Cells Derived from Rat Inferior Colliculi after Digestion with Papain
Source: PLoS One. 2013 Dec 12;8(12):e80490. doi: 10.1371/journal.pone.0080490 (PMC3861243; doi:10.1371/journal.pone.0080490)

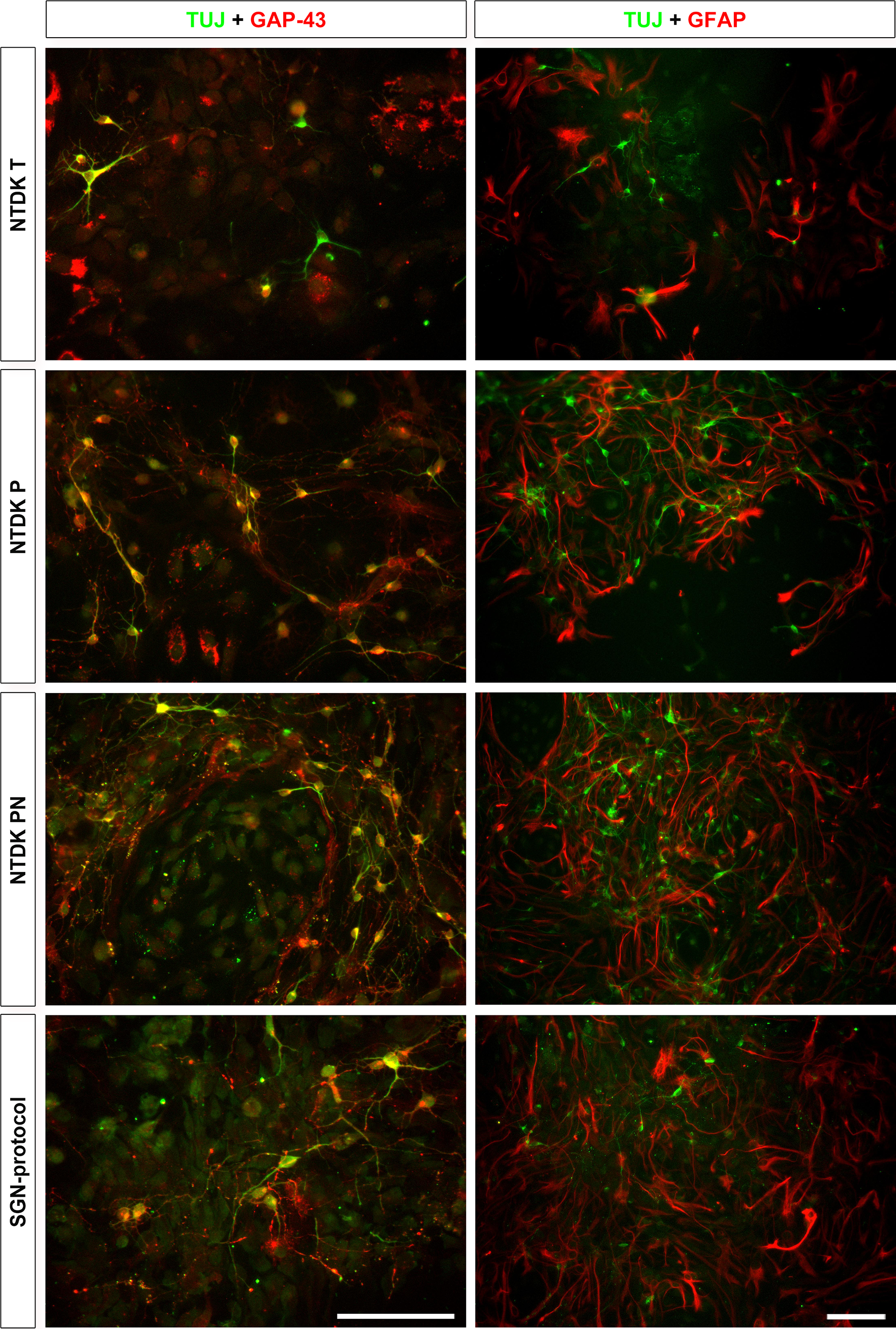

Supplement: Figure S1 — Immunocytochemical results for different dissociation protocols. After dissociation with different protocols (NTDK T, NTDK P, NTDK PN and SGN-protocol), herein presented cells were cultivated with Neuro Medium and fixed after 5 days. Cells were labelled with TUJ1 (green) and GFAP (red). Scale bar: 100 µm. (TIF) [file pone.0080490.s001.tif]

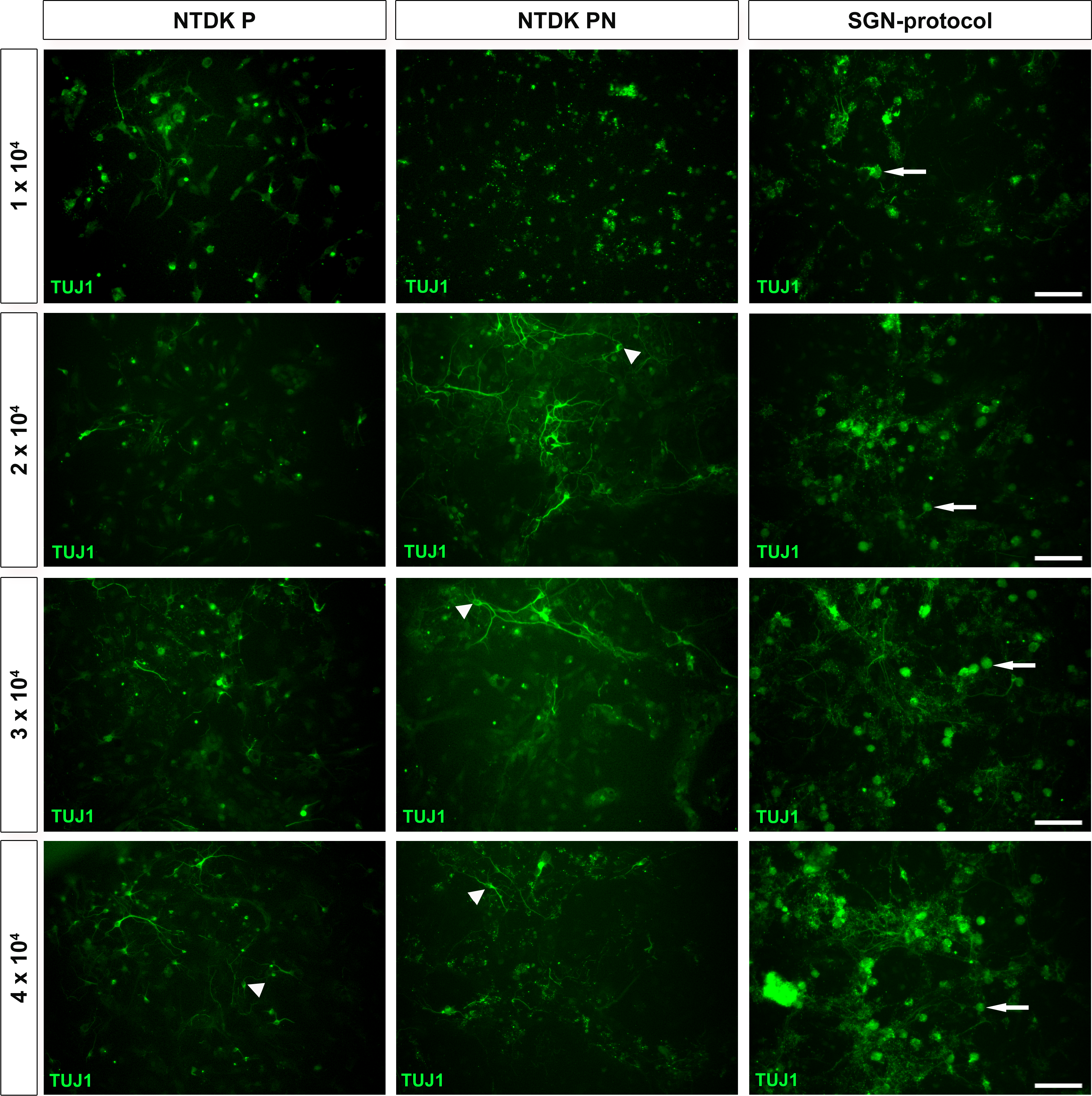

Supplement: Figure S2 — Fluorescence images of different seeding densities. Best images from the cells dissociated with three different methods (NTDK P: left column; NTDK PN: middle column and SGN-protocol: right column) seeded at four different densities (from 1×10^4^ cells/well: top row; to 4×10^4^ cells/well: bottom row) are depicted. TUJ1 labelled (green) images were shown. Arrows indicate the formation of cell clusters and arrowheads single neurons. Scale bar: 100 µm. (TIF) [file pone.0080490.s002.tif]

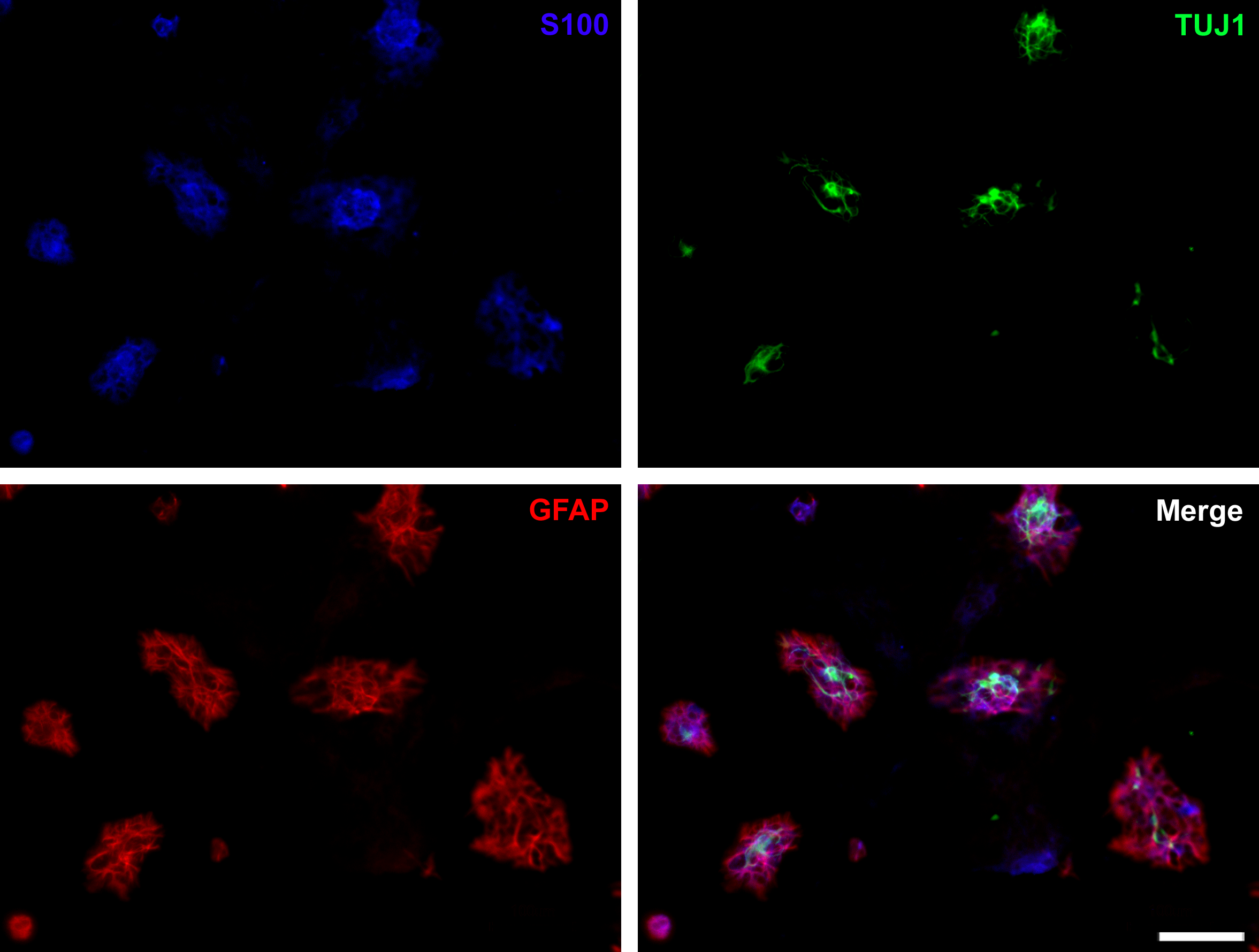

Supplement: Figure S3 — Triple staining of TUJ, GFAP, and S100. Both, anti-GFAP and anti-S100 antibodies stain astrocytes. In the triple staining (merge), astrocytes stained for GFAP (red) and for S100 (blue) seemed to be identical and clearly distinct from neurons (green). Scale bar: 100 µm. (TIF) [file pone.0080490.s003.tif]
