# Supplementary material for: Dissociated Neurons and Glial Cells Derived from Rat Inferior Colliculi after Digestion with Papain
Source: PLoS One. 2013 Dec 12;8(12):e80490. doi: 10.1371/journal.pone.0080490 (PMC3861243; doi:10.1371/journal.pone.0080490)
Supplement: Table S1 — (DOC) [file pone.0080490.s004.doc]

Supplemental Table 1a: Results obtained with MACS®Neuro Medium in the screening experiments

| MACS® Neuro Medium | **Confluency [%]** | | **Formation of cell clusters** | | **Cell debris** | | **Neuronal yield** | |
| --- | --- | --- | --- | --- | --- | --- | --- | --- |
|  | **2d** | **5d** | **2d** | **5d** | **2d** | **5d** | **2d** | **5d** |
| **NTDK T** | 20-30 ± 0 | 15-20 | + | (+) | + | + | (+) | - |
| **NTDK P** | 20 ± 10 | 30 | + | + | + | + | +(+) | +(+) |
| **NTDK PN** | 60 ± 10 | 60-70 | ++ | ++ | ++(+) | +(+) | +++ | +++ |
| **SGN** | 60 ± 10 | 60-70 | +(+) | ++ | +++ | ++ | +(+) | ++ |

Supplemental Table 1b: Results obtained with Panserin 401 in the screening experiments

| Panserin 401 | **Confluency [%]** | | **Formation of cell clusters** | | **Cell debris** | | **Neuronal yield** | |
| --- | --- | --- | --- | --- | --- | --- | --- | --- |
|  | **2d** | **5d** | **2d** | **5d** | **2d** | **5d** | **2d** | **5d** |
| **NTDK T** | 10-15 ± 0 | 10-15 | + | +(+) | + | + | (+) | + |
| **NTDK P** | 20 ± 10 | 15 | (+) - + | + | (+) - +(+) | + | (+) - ++ | +(+) |
| **NTDK PN** | 55 ± 5 | 30-40 | (+) - ++ | ++ | ++ - +++ | + | + - ++ | ++ |
| **SGN** | 45 ± 25 | 40-50 | + - ++ | +(+) | ++(+) - +++ | ++ | + - +(+) | +(+) |

*Confluency: rated confluence in per cent; optical evaluation of the whole well under bright-field conditions using a 40-fold magnification; all n (total number of wells) of one time point and kit were averaged to the confluency;*

*Cell debris, conglomerates, and neuronal yield*

*-: no debris, conglomerates or neurons;*

*+: poor amount; +(+): poor-middle;*

*++: middle amount; ++(+): middle-high;*

*+++: high amount*
